# 3-[(5-Chloro-2-hy­droxy­benzyl­idene)amino]-2-sulfanyl­idene-1,3-thia­zolidin-4-one

**DOI:** 10.1107/S1600536813016577

**Published:** 2013-06-19

**Authors:** Hakan Dal

**Affiliations:** a Department of Chemistry, Anadolu University, 26470 Eskişehir, Turkey

## Abstract

In the title compound, C_10_H_7_ClN_2_O_2_S_2_, the mean plane of the thioxo­thia­zolidine ring [maximum deviation = 0.032 (2) Å] is inclined to the benzene ring by 12.25 (4)°. There is a strong intra­molecular O—H⋯N hydrogen bond present. In the crystal, mol­ecules are linked *via* pairs of C—H⋯Cl hydrogen bonds, forming inversion dimers.

## Related literature

For general background to the chemistry, and pharmacological and biological activity of rhodanine and its derivatives, see: Raper (1985 ▶); Contello *et al.* (1994 ▶); Villain-Guillot *et al.* (2007 ▶); Yan *et al.* (2007 ▶); Kletzien *et al.* (1992 ▶). 
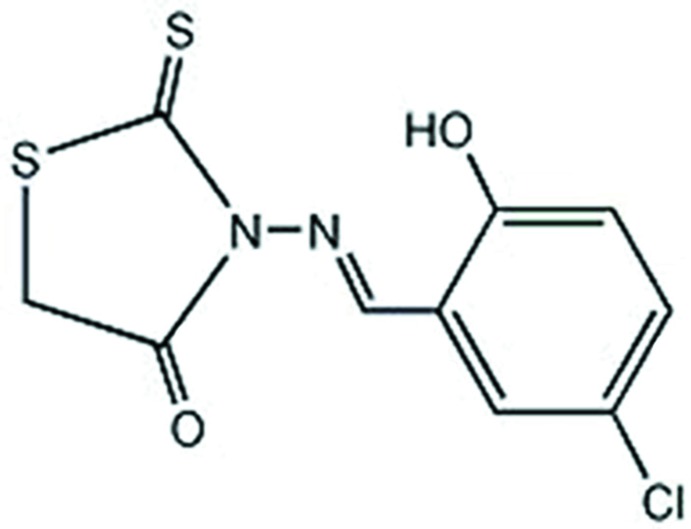


## Experimental

### 

#### Crystal data

C_10_H_7_ClN_2_O_2_S_2_
*M* *_r_* = 286.77
Monoclinic, 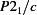

*a* = 9.8506 (3) Å
*b* = 10.0936 (3) Å
*c* = 12.1096 (4) Å
β = 110.409 (2)°
*V* = 1128.45 (6) Å^3^
*Z* = 4
Mo *K*α radiation
μ = 0.70 mm^−1^
*T* = 100 K
0.37 × 0.26 × 0.11 mm

#### Data collection

Bruker Kappa APEXII CCD area-detector diffractometer
Absorption correction: multi-scan (*SADABS*; Bruker, 2005 ▶) *T*_min_ = 0.783, *T*_max_ = 0.927
10564 measured reflections
2816 independent reflections
2427 reflections with *I* > 2σ(*I*)
*R*_int_ = 0.027

#### Refinement

*R*[*F* ^2^ > 2σ(*F* ^2^)] = 0.028
*wR*(*F* ^2^) = 0.081
*S* = 1.09
2816 reflections
162 parameters
H atoms treated by a mixture of independent and constrained refinement
Δρ_max_ = 0.45 e Å^−3^
Δρ_min_ = −0.31 e Å^−3^

### 

Data collection: *APEX2* (Bruker, 2007 ▶); cell refinement: *SAINT* (Bruker, 2007 ▶); data reduction: *SAINT*; program(s) used to solve structure: *SHELXS97* (Sheldrick, 2008 ▶); program(s) used to refine structure: *SHELXL97* (Sheldrick, 2008 ▶); molecular graphics: *ORTEP-3 for Windows* (Farrugia, 2012 ▶); software used to prepare material for publication: *WinGX* (Farrugia, 2012 ▶) and *PLATON* (Spek, 2009 ▶).

## Supplementary Material

Crystal structure: contains datablock(s) I, global. DOI: 10.1107/S1600536813016577/su2611sup1.cif

Structure factors: contains datablock(s) I. DOI: 10.1107/S1600536813016577/su2611Isup2.hkl

Supplementary material file. DOI: 10.1107/S1600536813016577/su2611Isup3.cml

Additional supplementary materials:  crystallographic information; 3D view; checkCIF report

## Figures and Tables

**Table 1:** Hydrogen-bond geometry (Å, °)

*D*—H⋯*A*	*D*—H	H⋯*A*	*D*⋯*A*	*D*—H⋯*A*
O2—H2⋯N2	0.75 (2)	1.97 (2)	2.6291 (19)	147 (3)
C9—H9*B*⋯Cl1^i^	0.99	2.81	3.7860 (19)	169

Symmetry code: (i) 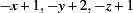.

**Fig. 1.**
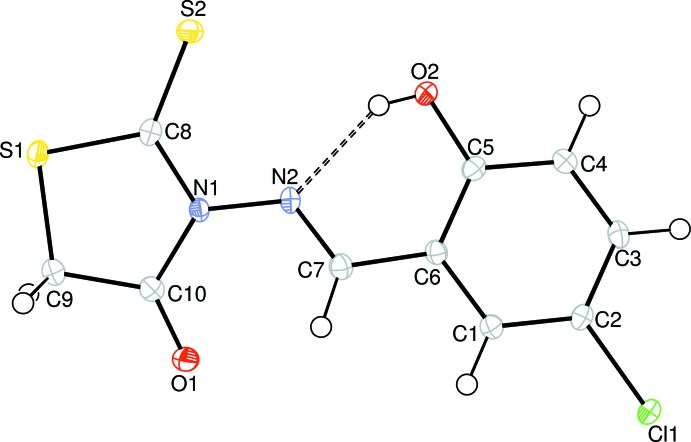
The molecular structure of the title molecule, with atom labelling. Displacement ellipsoids are drawn at the 50% probability level. The intramolecular O-H···N hydrogen bond is shown as a dashed line - see Table 1 for details.

## supplementary crystallographic information

### Comment

Rhodanine and its derivatives are used in a variety of applications ranging from industry to biochemistry and coordination chemistry. They have wide industrial applications as brightening additives in silver electroplating, intermediates in the syntheses of dyes, extreme-pressure lubricants and antioxidants as well as pharmacological (Contello *et al.*, 1994), and biological activities including antibacterial (Villain-Guillot *et al.*, 2007), antiviral (Yan *et al.*, 2007) and antidiabetical (Kletzien *et al.*, 1992). The interesting aspect of the chemistry of these compounds is their electron donating power to metal ions, which make them strong ligands in coordination compounds (Raper, 1985). herein we report on the crystal structure of the title rhodanine derivative.

In the molecule of the title compound (Fig. 1), the bond lengths and angles are generally within normal ranges. Ring B (S1/N1/C8–C10) is planar to within 0.032 (2) Å and is inclined to the benzene ring A (C1–C6) at a dihedral angle of 12.25 (4)°. Atoms Cl1, O2 and C7 are -0.0272 (4), -0.047 (2) and 0.052 (2) Å out of the plane of ring A, while atoms O1, S2 and N2 are 0.112 (2), -0.0327 (5) and 0.024 (2) Å displaced from the mean plane of ring B. The presence of the intramolecular O—H···N hydrogen bond (Table 1) forms a non-planar six-membered ring (O2/N2/H2/C5–C7), and contributes to the stabilization of the molecule.

In the crystal, molecules are linked via a pair of C-H···Cl hydrogen bonds forming inversion dimers (Table 1).

### Experimental

The title compound was prepared by the reaction of 2-hydroxy-5-chlorophenyl (0.63 g, 4 mmol) and N-amino rhodanine (0.50 g, 4 mmol) in methanol (50 ml) at room temperature. After stirring for 6 h, a fluffy yellow precipitate was obtained. The resulting crude solid was collected by filtration, dried and then purified by repeated recrystallization using methanol as solvent; yielding yellow block-like crystals.

### Refinement

Atoms H2 (for OH) and H7 (for methine) were located in a difference Fourier map and refined freely. The C-bound H-atoms were positioned geometrically with C—H = 0.95 and 0.99 Å for aromatic and methylene H-atoms, respectively, and constrained to ride on their parent atoms, with *U*_iso_(H) = 1.2 × *U*_eq_(C).

### Crystal data

**Table:**

C_10_H_7_ClN_2_O_2_S_2_	*F*(000) = 584
*M**_r_* = 286.77	*D*_x_ = 1.688 Mg m^−^^3^
Monoclinic, *P*2_1_/*c*	Mo *K*α radiation, λ = 0.71073 Å
Hall symbol: -P 2ybc	Cell parameters from 4301 reflections
*a* = 9.8506 (3) Å	θ = 2.2–28.3°
*b* = 10.0936 (3) Å	µ = 0.70 mm^−^^1^
*c* = 12.1096 (4) Å	*T* = 100 K
β = 110.409 (2)°	Block, yellow
*V* = 1128.45 (6) Å^3^	0.37 × 0.26 × 0.11 mm
*Z* = 4	

### Data collection

**Table:**

Bruker Kappa APEXII CCD area-detector diffractometer	2816 independent reflections
Radiation source: fine-focus sealed tube	2427 reflections with *I* > 2σ(*I*)
Graphite monochromator	*R*_int_ = 0.027
φ and ω scans	θ_max_ = 28.4°, θ_min_ = 2.2°
Absorption correction: multi-scan (*SADABS*; Bruker, 2005)	*h* = −13→13
*T*_min_ = 0.783, *T*_max_ = 0.927	*k* = −13→13
10564 measured reflections	*l* = −16→16

### Refinement

**Table:**

Refinement on *F*^2^	Primary atom site location: structure-invariant direct methods
Least-squares matrix: full	Secondary atom site location: difference Fourier map
*R*[*F*^2^ > 2σ(*F*^2^)] = 0.028	Hydrogen site location: inferred from neighbouring sites
*wR*(*F*^2^) = 0.081	H atoms treated by a mixture of independent and constrained refinement
*S* = 1.09	*w* = 1/[σ^2^(*F*_o_^2^) + (0.0379*P*)^2^ + 0.6866*P*] where *P* = (*F*_o_^2^ + 2*F*_c_^2^)/3
2816 reflections	(Δ/σ)_max_ = 0.001
162 parameters	Δρ_max_ = 0.45 e Å^−^^3^
0 restraints	Δρ_min_ = −0.31 e Å^−^^3^

### Special details

**Table:**

Geometry. All esds (except the esd in the dihedral angle between two l.s. planes) are estimated using the full covariance matrix. The cell esds are taken into account individually in the estimation of esds in distances, angles and torsion angles; correlations between esds in cell parameters are only used when they are defined by crystal symmetry. An approximate (isotropic) treatment of cell esds is used for estimating esds involving l.s. planes.
Refinement. Refinement of F^2^ against ALL reflections. The weighted R-factor wR and goodness of fit S are based on F^2^, conventional R-factors R are based on F, with F set to zero for negative F^2^. The threshold expression of F^2^ > 2sigma(F^2^) is used only for calculating R-factors(gt) etc. and is not relevant to the choice of reflections for refinement. R-factors based on F^2^ are statistically about twice as large as those based on F, and R- factors based on ALL data will be even larger.

### Fractional atomic coordinates and isotropic or equivalent isotropic displacement parameters (Å^2^)

**Table:**

	*x*	*y*	*z*	*U*_iso_*/*U*_eq_	
S1	−0.07187 (4)	1.08989 (5)	0.26371 (4)	0.01535 (11)	
S2	−0.06151 (4)	1.23019 (5)	0.48023 (4)	0.01547 (11)	
O1	0.31445 (14)	0.95179 (13)	0.40194 (11)	0.0179 (3)	
O2	0.25301 (14)	1.25062 (13)	0.75822 (11)	0.0163 (3)	
H2	0.221 (3)	1.231 (2)	0.695 (2)	0.032 (7)*	
N1	0.15074 (15)	1.07839 (14)	0.45427 (12)	0.0117 (3)	
N2	0.23170 (15)	1.11112 (15)	0.56955 (12)	0.0129 (3)	
C1	0.55038 (18)	1.01067 (18)	0.80910 (14)	0.0139 (3)	
H1	0.5808	0.9404	0.7710	0.017*	
C2	0.63231 (17)	1.04465 (18)	0.92291 (14)	0.0133 (3)	
C3	0.59104 (18)	1.14826 (18)	0.98024 (14)	0.0147 (3)	
H3	0.6496	1.1723	1.0582	0.018*	
C4	0.46398 (19)	1.21576 (18)	0.92266 (15)	0.0155 (3)	
H4	0.4350	1.2861	0.9617	0.019*	
C5	0.37768 (18)	1.18186 (18)	0.80794 (14)	0.0135 (3)	
C6	0.42240 (17)	1.07939 (17)	0.74940 (14)	0.0123 (3)	
C7	0.34218 (18)	1.04111 (18)	0.62815 (15)	0.0143 (3)	
H7	0.378 (2)	0.966 (2)	0.5974 (19)	0.019 (5)*	
C8	0.01423 (18)	1.13498 (17)	0.40910 (14)	0.0129 (3)	
C9	0.07577 (19)	0.98704 (19)	0.25954 (15)	0.0164 (4)	
H9A	0.0442	0.8935	0.2455	0.020*	
H9B	0.1092	1.0160	0.1952	0.020*	
C10	0.19599 (19)	1.00064 (17)	0.37653 (15)	0.0136 (3)	
Cl1	0.79051 (4)	0.95632 (4)	0.99429 (3)	0.01531 (11)	

### Atomic displacement parameters (Å^2^)

**Table:**

	*U*^11^	*U*^22^	*U*^33^	*U*^12^	*U*^13^	*U*^23^
S1	0.0133 (2)	0.0190 (2)	0.0114 (2)	−0.00020 (16)	0.00125 (16)	−0.00067 (16)
S2	0.0143 (2)	0.0150 (2)	0.0173 (2)	0.00122 (16)	0.00585 (16)	−0.00154 (16)
O1	0.0186 (6)	0.0198 (7)	0.0154 (6)	0.0049 (5)	0.0061 (5)	−0.0001 (5)
O2	0.0158 (6)	0.0176 (7)	0.0128 (6)	0.0054 (5)	0.0016 (5)	−0.0009 (5)
N1	0.0130 (7)	0.0106 (7)	0.0098 (6)	−0.0006 (5)	0.0020 (5)	−0.0005 (5)
N2	0.0131 (6)	0.0146 (8)	0.0098 (6)	−0.0020 (6)	0.0025 (5)	−0.0006 (5)
C1	0.0142 (8)	0.0152 (9)	0.0123 (8)	0.0004 (7)	0.0048 (6)	−0.0003 (6)
C2	0.0111 (7)	0.0147 (9)	0.0126 (8)	−0.0009 (6)	0.0023 (6)	0.0017 (6)
C3	0.0149 (8)	0.0172 (9)	0.0111 (7)	−0.0027 (7)	0.0033 (6)	−0.0020 (7)
C4	0.0165 (8)	0.0157 (9)	0.0144 (8)	0.0008 (7)	0.0054 (7)	−0.0019 (7)
C5	0.0126 (7)	0.0142 (9)	0.0137 (8)	0.0002 (7)	0.0047 (6)	0.0022 (6)
C6	0.0135 (8)	0.0112 (9)	0.0119 (8)	−0.0017 (6)	0.0039 (6)	−0.0006 (6)
C7	0.0152 (8)	0.0138 (9)	0.0134 (8)	−0.0011 (7)	0.0044 (6)	−0.0004 (7)
C8	0.0126 (8)	0.0129 (8)	0.0122 (7)	−0.0021 (6)	0.0030 (6)	0.0019 (6)
C9	0.0172 (8)	0.0186 (10)	0.0127 (8)	0.0002 (7)	0.0041 (7)	−0.0023 (7)
C10	0.0177 (8)	0.0110 (8)	0.0126 (8)	0.0000 (7)	0.0062 (6)	0.0022 (6)
Cl1	0.01286 (19)	0.0175 (2)	0.01301 (19)	0.00297 (15)	0.00127 (15)	0.00100 (15)

### Geometric parameters (Å, º)

**Table:**

S1—C8	1.7277 (17)	C3—H3	0.9500
S1—C9	1.8018 (18)	C4—C3	1.381 (2)
S2—C8	1.6341 (17)	C4—C5	1.395 (2)
O1—C10	1.204 (2)	C4—H4	0.9500
O2—C5	1.355 (2)	C5—C6	1.409 (2)
O2—H2	0.74 (3)	C7—N2	1.284 (2)
N1—N2	1.3848 (19)	C7—C6	1.456 (2)
N1—C8	1.386 (2)	C7—H7	0.97 (2)
N1—C10	1.412 (2)	C9—C10	1.503 (2)
C1—C6	1.399 (2)	C9—H9A	0.9900
C1—H1	0.9500	C9—H9B	0.9900
C2—C1	1.376 (2)	Cl1—C2	1.7410 (17)
C2—C3	1.392 (2)		
			
C8—S1—C9	93.79 (8)	C4—C5—C6	119.50 (16)
C5—O2—H2	109 (2)	C1—C6—C5	119.18 (15)
N2—N1—C8	115.97 (13)	C1—C6—C7	117.63 (15)
N2—N1—C10	126.87 (14)	C5—C6—C7	123.19 (16)
C8—N1—C10	117.03 (14)	N2—C7—C6	117.94 (16)
C7—N2—N1	120.33 (15)	N2—C7—H7	125.1 (13)
C2—C1—C6	120.14 (16)	C6—C7—H7	116.9 (13)
C2—C1—H1	119.9	S2—C8—S1	122.68 (10)
C6—C1—H1	119.9	N1—C8—S1	110.95 (12)
C1—C2—C3	121.03 (16)	N1—C8—S2	126.36 (13)
C1—C2—Cl1	118.72 (13)	S1—C9—H9A	110.2
C3—C2—Cl1	120.24 (13)	S1—C9—H9B	110.2
C2—C3—H3	120.3	C10—C9—S1	107.42 (12)
C4—C3—C2	119.31 (16)	C10—C9—H9A	110.2
C4—C3—H3	120.3	C10—C9—H9B	110.2
C3—C4—C5	120.79 (16)	H9A—C9—H9B	108.5
C3—C4—H4	119.6	O1—C10—N1	124.09 (16)
C5—C4—H4	119.6	O1—C10—C9	125.35 (16)
O2—C5—C4	117.39 (15)	N1—C10—C9	110.55 (14)
O2—C5—C6	123.12 (15)		
			
C9—S1—C8—S2	−177.82 (12)	Cl1—C2—C1—C6	179.78 (13)
C9—S1—C8—N1	1.29 (13)	C1—C2—C3—C4	1.5 (3)
C8—S1—C9—C10	−3.74 (13)	Cl1—C2—C3—C4	−179.02 (13)
C8—N1—N2—C7	163.12 (15)	C5—C4—C3—C2	−0.5 (3)
C10—N1—N2—C7	−21.1 (2)	C3—C4—C5—O2	178.56 (15)
N2—N1—C8—S1	178.09 (11)	C3—C4—C5—C6	−1.4 (3)
N2—N1—C8—S2	−2.8 (2)	O2—C5—C6—C1	−177.81 (15)
C10—N1—C8—S1	1.86 (18)	O2—C5—C6—C7	2.7 (3)
C10—N1—C8—S2	−179.07 (13)	C4—C5—C6—C1	2.1 (2)
N2—N1—C10—O1	0.0 (3)	C4—C5—C6—C7	−177.44 (16)
N2—N1—C10—C9	179.45 (15)	C6—C7—N2—N1	−179.40 (14)
C8—N1—C10—O1	175.75 (16)	N2—C7—C6—C1	−174.04 (16)
C8—N1—C10—C9	−4.8 (2)	N2—C7—C6—C5	5.5 (3)
C2—C1—C6—C5	−1.1 (2)	S1—C9—C10—O1	−175.28 (15)
C2—C1—C6—C7	178.51 (15)	S1—C9—C10—N1	5.26 (17)
C3—C2—C1—C6	−0.8 (3)		

### Hydrogen-bond geometry (Å, º)

**Table:**

*D*—H···*A*	*D*—H	H···*A*	*D*···*A*	*D*—H···*A*
O2—H2···N2	0.75 (2)	1.97 (2)	2.6291 (19)	147 (3)
C9—H9*B*···Cl1^i^	0.99	2.81	3.7860 (19)	169

Symmetry code: (i) −*x*+1, −*y*+2, −*z*+1.

## References

[bb1] Bruker (2005). *SADABS* Bruker AXS Inc., Madison, Wisconsin, USA.

[bb2] Bruker (2007). *APEX2* and *SAINT* Bruker AXS Inc., Madison, Wisconsin, USA.

[bb3] Contello, B. C. C., Cawhorne, M. A., Haigh, D., Hindley, R. M., Smith, S. A. & Thurlby, P. L. (1994). *Bioorg. Med. Chem. Lett.* **4**, 1181–1184.

[bb4] Farrugia, L. J. (2012). *J. Appl. Cryst.* **45**, 849–854.

[bb5] Kletzien, R. F., Clarke, S. D. & Ulrich, R. G. (1992). *Mol. Pharmacol.* **41**, 393–398. 1538716

[bb6] Raper, E. S. (1985). *Coord. Chem. Rev.* **61**, 115–184.

[bb7] Sheldrick, G. M. (2008). *Acta Cryst.* A**64**, 112–122. 10.1107/S0108767307043930 18156677

[bb8] Spek, A. L. (2009). *Acta Cryst.* D**65**, 148–155. 10.1107/S090744490804362X PMC2631630 19171970

[bb9] Villain-Guillot, P., Gualtieri, M., Bastide, L., Roquet, F., Martinez, J., Amblard, M., Pugniere, M. & Leonetti, J. P. (2007). J. Med. Chem. 50, 4195–4204. 10.1021/jm0703183 17665895

[bb10] Yan, S., Larson, G., Wu, J. Z., Applby, T., Ding, Y., Hamatake, R., Hong, Z. & Yao, N. (2007). *Bioorg. Med. Chem. Lett.* **17**, 63–67. 10.1016/j.bmcl.2006.09.095 17049849

